# *Limnofasciculus baicalensis* gen. et sp. nov. (Coleofasciculaceae, Coleofasciculales): A New Genus of Cyanobacteria Isolated from Sponge Fouling in Lake Baikal, Russia

**DOI:** 10.3390/microorganisms11071779

**Published:** 2023-07-09

**Authors:** Ekaterina Sorokovikova, Irina Tikhonova, Peter Evseev, Andrey Krasnopeev, Igor Khanaev, Sergey Potapov, Anna Gladkikh, Ivan Nebesnykh, Olga Belykh

**Affiliations:** 1 Limnological Institute of the Siberian Branch of the Russian Academy of Sciences, 3 Ulan-Batorskaya Str., Irkutsk 664033, Russia; 2 Shemyakin-Ovchinnikov Institute of Bioorganic Chemistry of the Russian Academy of Sciences, 16/10 Miklukho-Maklaya Str., GSP-7, Moscow 117997, Russia; 3 Saint-Petersburg Pasteur Institute, 14 Mira Str., Saint-Petersburg 197101, Russia

**Keywords:** freshwater benthic cyanobacteria, Lake Baikal, *Limnofasciculus*, *Symplocastrum*, cyanobacterial phylogeny, cyanobacterial taxonomy, biosynthetic gene clusters, bioactive metabolites

## Abstract

The proliferation of benthic cyanobacteria has been observed in Lake Baikal since 2011 and is a vivid manifestation of the ecological crisis occurring in the littoral zone. The cyanobacterium *Symplocastrum* sp. has formed massive fouling on all types of benthic substrates, including endemic Baikal sponges. The strain BBK-W-15 (=IPPAS B-2062^T^), which was isolated from sponge fouling in 2015, was used for further taxonomic determination. A polyphasic approach revealed that it is a cryptic taxon of cyanobacteria. Morphological evaluation of the strain indicated the presence of cylindrical filaments with isodiametric cells enclosed in individual sheaths and coleodesmoid false branching. Strain ultrastructure (fascicular thylakoids and type C cell division) is characteristic of the Microcoleaceae and Coleofasciculaceae families. An integrated analysis that included 16S rRNA gene phylogeny, conserved protein phylogeny and whole-genome comparisons indicated the unique position of BBK-W-15, thus supporting the proposed delineation of the new genus *Limnofasciculus*. Through characterisation by morphology, 16S, ITS and genomic analysis, a new cyanobacterium of the family Coleofasciculaceae *Limnofasciculus baicalensis* gen. et sp. nov. was described.

## 1. Introduction

Lake Baikal is one of the largest lakes in the world, containing approximately 20% of the Earth’s fresh liquid surface water, and it is a UNESCO World Natural Heritage site. Baikal is one of the most ancient lakes, the age of which is estimated at 25 million years, and it provides a natural laboratory for studying the evolution and processes of endemic speciation [1]. The algoflora of the lake includes 569 species and 162 varieties of algae, of which 35% are endemic [2]. In bottom phytocenoses, the proportion of endemic species reaches 69% [3].

Since 2011, an ecological crisis has been observed in the coastal zone of Lake Baikal, which has led to dramatic changes in key links within the lake’s ecosystem [4]. There has been a shift in the species composition of shallow phytoplankton, from the “Baikal complex” of diatoms to small-celled green algae and euglenophytes, which are indicators of organic pollution [5]. The proliferation of benthic cyanobacteria and the occupation of the habitats of endemic *Draparnaldioides* by *Spirogyra* spp. has changed the usual vegetation zones and species composition of phytobenthos and led to the disruption of unique aquascapes [4,6,7]. Another pronounced harmful manifestation of the crisis has been the mass disease affecting endemic Baikal sponges, with their surface often being covered with biofilms and the fouling of filamentous cyanobacteria [8,9,10,11,12]. The dominant species in sponge fouling has often been cyanobacterium, which the authors initially identified as *Symplocastrum* sp. It has formed massive red–brown bushes on stones, sheer rocks and endemic sponges [9,12,13], and it has been found in plankton in the form of photogranules [14]. The authors assumed that *Symplocastrum* sp. was recorded as *Schizothrix* sp. in earlier studies of the phytobenthos of Lake Baikal [3], but found significant differences in the ecology and morphology of these species, requiring a description and clarification of the taxonomic position of *Symplocastrum* sp.

In 1898, O. Kirchner singled out the subgenus *Symplocastrum* from *Schizothrix* and raised it to genus level, though this taxonomic scheme was not used for a long time in most classical monographies [15]. It was only in 2005 that *Symplocastrum* was considered to be a valid genus of the subfamily Microcoleoideae of the family Phormidiaceae, later being reclassified as part of the family Microcoleaceae [16,17].

Representatives of the genus *Symplocastrum* are characterised by filaments grouped into fascicles and arranged in abundantly branching tuft-like thalli. Sheaths are wide, colourless and branched, and they contain one or multiple parallel-arranged trichomes. The length of the cells is mostly greater than their width. The genus *Symplocastrum* includes 19 species, which leads to an attached lifestyle; freshwater species are epiliths, epiphytes and foulers of wood substrates [18,19]. Using cultures isolated from North American desert soils, a genetic study of the species *S*. *californicum*, *S*. *flechtnerae* and *S*. *torsivum* was carried out, and for the latter species, a genome was obtained [20]. It has been confirmed that the genus *Symplocastrum* is unique and phylogenetically unrelated to *Schizothrix*, in which it genus was previously included [21]. It is, however, difficult to estimate the genetic homogeneity of the genus *Symplocastrum* as a whole, because the sequences of numerous species from freshwater and tropical ecotopes are not available in the GenBank database.

The morphology of thalli and trichomes of both natural samples from Lake Baikal and the isolated strain have been shown to correspond to the *Symplocastrum* genus Microcoleaceae family [19]. A preliminary analysis, however, indicated markedly low rRNA and genomic similarities between the novel strain and other cyanobacterial sequences, indicating that the novel strain represents a cryptic genus. Cryptic genera are especially difficult to define in terms of their taxonomy because they do not meet the commonly used criteria for describing taxa. Morphological markers of cyanobacterial cryptotaxa are unclear, and, sometimes, cytological (ultrastructural) or ecological differences are used as auxiliary characters, though the main basis for their identification is molecular data [16,22].

In this study, the BBK-W-15 strain was isolated from the fouling of a diseased sponge collected in Southern Baikal, near the Bolshiye Koty settlement. The application of a polyphasic method based on morphology, cell ultrastructure and genome analysis showed that it constitutes a new genus of cyanobacteria. Thus, the new genus *Limnofasciculus* gen. nov. and the type species *Limnofasciculus baicalensis* sp. nov. have been described.

## 2. Materials and Methods

### 2.1. Sampling

**Figure 1:**
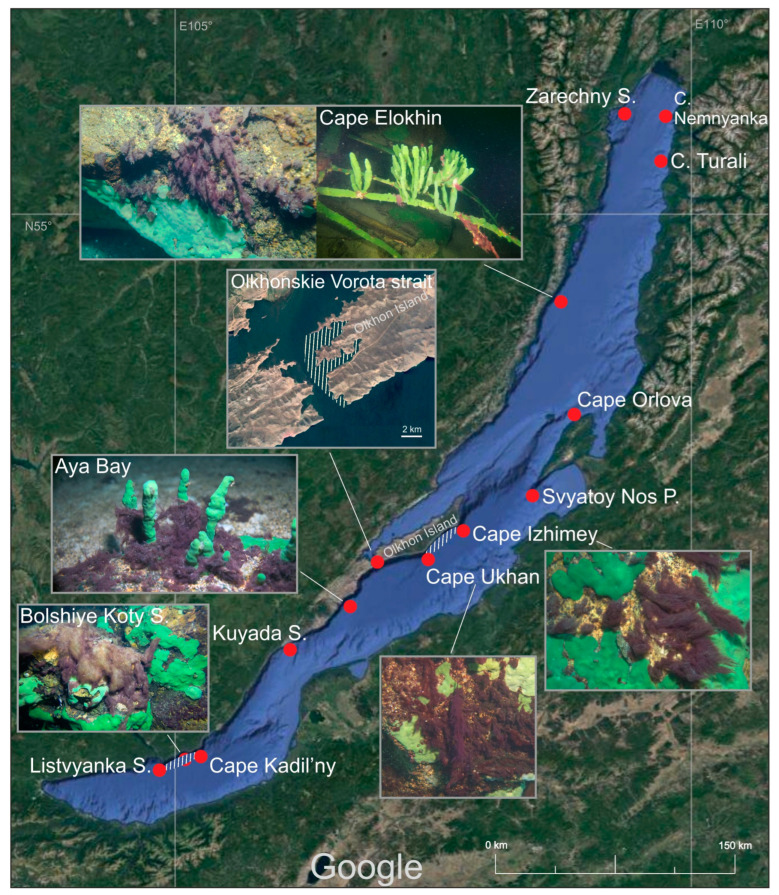
Map of the occurrence of the *Limnofasciculus baicalensis* gen. et sp. nov. in Lake Baikal, Russia, according to the sampling data gathered by research divers in the period 2015–2021, all seasons, and at depths of 3–20 m. Abbreviations and designations: S.—settlement; C.—cape; P.—peninsula; red points—sampling sites where the species was found; white shading—areas of the littoral with the ubiquitous occurrence of the species. Imagery © 2021 NASA, TerraMetrics, Map data © 2021 INEGI.

Samples were taken by scuba divers at depths of 3–20 m, from 2015 to 2021, in all seasons, and along the entire coast of Lake Baikal. The stations and areas of the littoral area in which the species was recorded are shown in Figure 1. Before sampling, divers surveyed, measured the length of and photographed the fouling with a Sony A7 camera (Tokyo, Japan), GoPro HERO 3+ or GoPro HERO 7 video camera (GoPro Inc., San Mateo, CA, USA), which was fixed in a protective waterproof box equipped with additional artificial light Ikelite PRO-2800 (Indianapolis, IN, USA). Next, on board the research vessel, samples for microscopic examination were treated with 4% formaldehyde (final concentration). For cultivation, pieces of sponges and stones with fouling were placed in containers of Baikal water and stored at +4–8 °C until delivery to the laboratory.

### 2.2. Collection

The strain was isolated from the fouling of the sponge *Lubomirskia baikalensis* and collected in February 2015 near the Bolshiye Koty settlement (51°54′07, 3″ N, 105°06′20, 2″ E) at a depth of 9 m. Before cultivation, the fouling fragments were washed twice with sterile Z-8 medium [23] and placed onto agar-solidified Z-8 medium in sterile Petri dishes. Enrichment dishes were maintained in a growth chamber at 12 °C under an Aqua-Glo aquarium plant lamp (Hagen, Germany) using a 16:8 h light:dark cycle with a photon flux density of 14 μmol m^−2^ s^−1^. After 8 weeks of growth, single filaments were picked from enrichment dishes and placed in separate tubes with liquid Z-8 medium supplemented by 0.4 µg mL^−1^ cycloheximide (BioChemica, PanReac Applichem, Darmstadt, Germany) to prevent the growth of eukaryotic algae. Finally, a unialgal strain was isolated and transferred into several 100 mL flasks that contained 50 mL of Z-8 medium. This strain was designated BBK-W-15 and stored in the culture collection of the Laboratory of Aquatic Microbiology of the Limnological Institute of SB RAS, Irkutsk, Russia. The strain BBK-W-15 was deposited in the collection of microalgae and cyanobacteria of the Timiryazev Institute of Plant Physiology RAS, Moscow, Russia (=IPPAS B-2062^T^).

A herbarium specimen was prepared from a subsample of BBK-W-15 by drying the colony on a 0.2 µm membrane (Millipore, Burlington, MA, USA). The filter containing the cyanobacterial biofilm was submitted to the IRKU Herbarium (Irkutsk State University, Irkutsk, Russia) and is available under the number IRKU092121.

### 2.3. Microscopic Analysis

Morphological variability in the population was evaluated from cultured samples and 4% formaldehyde-treated field material using an Axio Imager (Carl Zeiss, Jena, Germany) microscope equipped with an AxioCam MRc5 camera. Taxonomic characteristics, such as trichome width and cell length, were measured in 200 cells for natural samples, as well as for the strain using Image-Pro Plus 6.0 software (http://www.mediacy.com, accessed on 15 April 2022). The strain was identified according to [19]. A macro-photo of the colonies was taken using a Coolpix S6800 camera (Nikon, Tokyo, Japan). Digital drawings were made using the Krita 5.0.6 program (https://krita.org/en/, accessed on 15 April 2022) based on microscopic images.

For analysis using a scanning electron microscope (SEM), thin sterile glasses for fouling were introduced into the liquid medium Z-8 with a culture of cyanobacteria. Next, the biofilm samples on the glasses were treated with 2% formaldehyde and dehydrated through a series of ethanol, increasing the concentration. After drying at 40 °C, the samples were coated in gold using a Balzers SCD 004 sputter-coater (Bal-Tec AG, Balzers, Liechtenstein) and examined using a SEM Quanta 200 (FEI Co., Hillsboro, OR, USA).

Sample preparation for transmission electron microscopy (TEM) was carried out as described in [24]. Sections were examined using a Leo 906E transmission electron microscope (Carl Zeiss, Jena, Germany).

### 2.4. DNA Extraction and Genome Sequencing

Genomic DNA was isolated from the culture using enzymatic lysis–lysozyme (1 µg mL^−1^) (Roche, Basel, Switzerland), proteinase K (1 µg mL^−1^) (Thermo Scientific, Waltham, MA, USA) and sodium dodecyl sulfate (1 µg mL^−1^) (VWR Life Science, Radnor, PA, USA), followed by phenol and chloroform (Medigen, Novosibirsk, Russia) extraction [25]. Libraries were prepared using the Nextera DNA Flex library prep kit (Illumina Inc., San Diego, CA, USA) according to the manufacturer’s instructions. Sequencing was performed via a MiSeq instrument using MiSeq V3 chemistry, achieving 2 × 300 nucleotide base pair (bp) reads. De novo genome assembly was performed using SPAdes 3.12 with default settings [26] and deposited in GenBank under accession #JAMZMM000000000. Binning was performed using MetaBAT 2 [27]. The heterogeneity check and assessment of completeness and contamination scores were carried out using CheckM [28].

### 2.5. Genome Annotation and Proteome Analysis

The assembled genome was annotated using the Prokaryotic Genome Annotation Pipeline (PGAP) [29] via NCBI services (https://www.ncbi.nlm.nih.gov/, accessed on 19 April 2023). Clusters of orthologous groups of proteins (COGs) were identified using the eggNOG-mapper 2 server [30] using genome sequences and by applying the default settings. Biosynthetic gene clusters (BGCs) in studied genomes were annotated using antiSMASH v. 6.1.1 [31] on a local machine that had the following parameters: ‘--genefinding-tool prodigal--fullhmmer--clusterhmmer--tigrfam--asf--cc-mibig--cb-general--cb-subclusters--cb-knownclusters--pfam2go--rre–html-start-compact’.

### 2.6. Average Nucleotide Identity Calculations and Phylogenetic Analysis

Cyanobacterial complete and draft genome sequences were downloaded from the NCBI Genome database, and 16S rDNA and internal transcribing spacer (ITS) sequences were downloaded from the NCBI Nucleotide database. The relevance of species names was checked in Algaebase (https://www.algaebase.org, accessed on 19 April 2023). The average nucleotide identity (ANI) clustered heatmap was obtained using orthoANIu [32] and Bio-NJ clustering [33]. Alignments of 16S rDNA sequences were obtained using MAFFT 7.48 via default settings and the L-INS-i algorithm [34,35]. A 16S rDNA phylogenetic tree was constructed using RAxML-NG 1.1.0 [36] and the raxmlGUI 2.0.10 graphic interface [37] with ‘--tree rand {10}--bs-trees 1000′ settings, as well as by applying the best nucleotide substitution model found using ModelTest-NG 0.1.7 [38]. Alignment of concatenated sequences of orthologous proteins was performed via PhyloPhlAn 3.0, which applied ‘-d phylophlan-diversity medium-f supermatrix_aa.cfg‘ settings [39]. The tree was constructed using RAxML-NG based on ‘-tree rand {1}-bs-trees 200′ settings. The robustness of the RAxML-NG trees was assessed using bootstrapping and calculations of transfer bootstrap estimation (TBE) support [40]. The NCBI accession numbers of all sequences used to construct the phylogenetic tree are given in Table S1.

## 3. Results

### 3.1. Distribution

**Figure 2:**
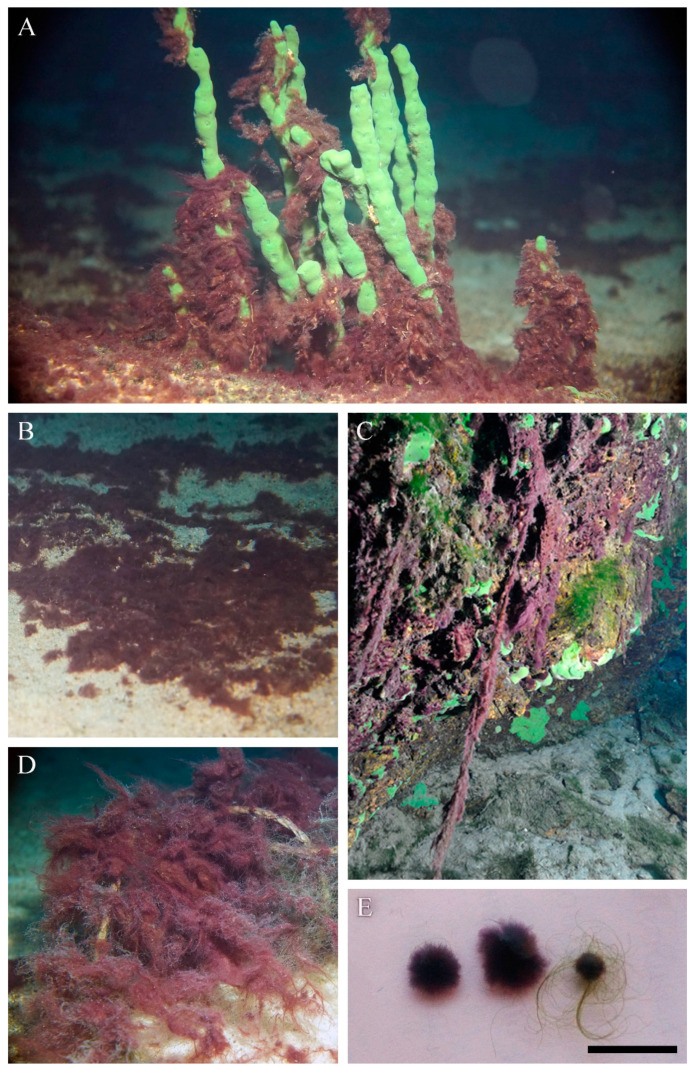
Ecology of cyanobacteria *Limnofasciculus baicalensis* gen. et sp. nov. in Lake Baikal. (**A**–**D**) Variety of fouling substrates: stone and branching sponge *Lubomirskia baikalensis* (**A**), sand (**B**), stone and encrusting sponge *Baikalospongia* sp. (**C**), fishing net (**D**). (**E**) Shows photogranules in plankton. Scale bar = 1 cm.

The proliferation of *Symplocastrum* sp. has been observed in Baikal since 2015. The species is found at depths in the range 3–10 (20) m along the western shore of the lake, from the southern tip to the Zarechny settlement in the north basin; along the eastern shore, from the southern tip of the Svyatoy Nos Peninsula to Nemnyanka Cape; and along the southern and eastern coasts of Olkhon Island (Figure 1). Particularly massive fouling was observed from June to October in Aya Bay, as well as near Listvyanka and Bolshiye Koty and north of Ukhan Cape on the seaward side of Olkhon Island (Figure S1). It occurs on stony substrates, sandy substrates, encrusting and branching sponges, and submerged objects, such as fishing nets, wood and steel (anchors and various steelworks) (Figure 2A–D). In autumn, during strong wind–wave activity, the species was observed in plankton as having a width of 0.5–1.5 cm photogranules (Figure 2E).

### 3.2. Morphological Investigation of Natural Samples and Strain

**Figure 3:**
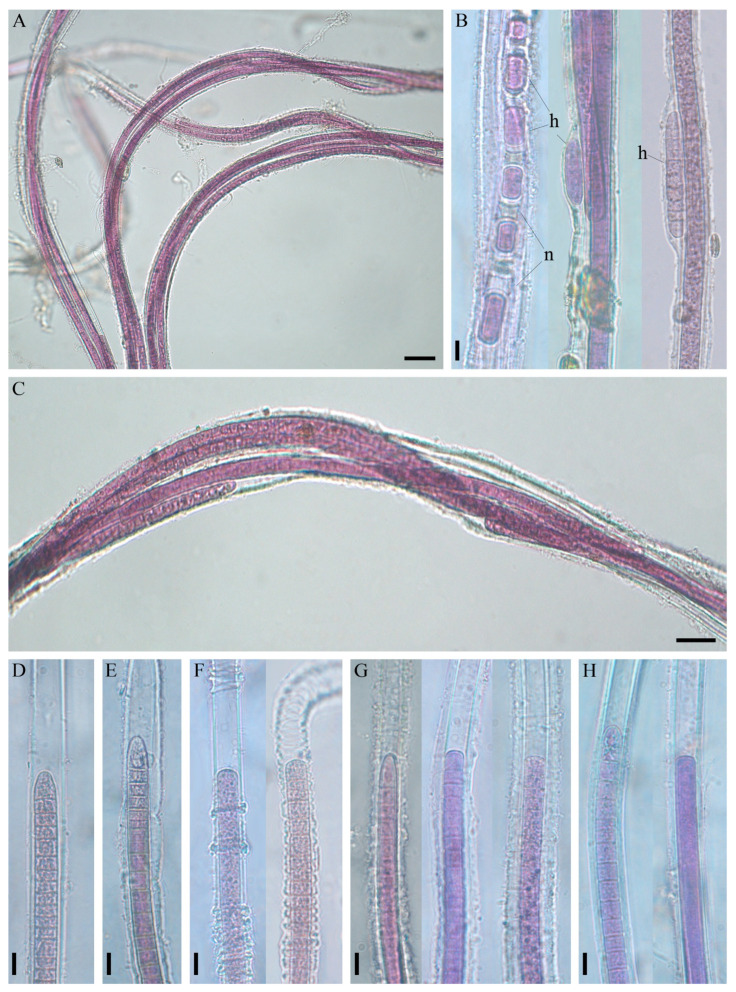
Morphology of *Limnofasciculus baicalensis* gen. et sp. nov. from benthic foulings. (**A**–**C**) Structure of fascicles. (**B**) Formation of hormogonia (h) via means of necridic cells (n) and germinating hormogonium within the mother sheath. (**D**–**H**) Different types of sheath: thin (**D**), widened (**E**), transversely folded (**F**), thick, diffluent at the margin (**G**), thick, lamellated (**H**). Scale bar (**A**) = 50 μm; (**B**,**D**–**H**) = 10 μm; (**C**) = 20 μm.

**Figure 4:**
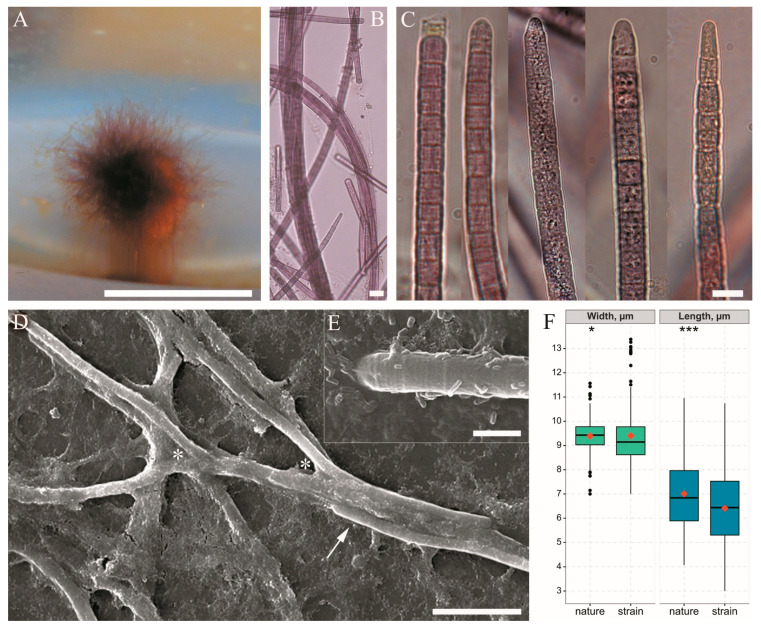
Morphology of *Limnofasciculus baicalensis* gen. et sp. nov. strain BBK-W-15. (**A**) A spherical rhizoidal colony in a liquid medium; (**B**) the light microscopy of the colony; (**C**) different types of apical cell; (**D**) scanning electron micrographs strain that show filaments aggregated to fascicle (arrow), which are often pseudoranched (asterisks); (**E**) the end of a trichome enclosed in a sheath with numerous bacteria in the sheath mucus; (**F**) the cell size obtained by measuring microphotographs of natural samples and strain, in which the red dot indicates the mean value, the line indicates the median value, the box indicates the interquartile range from Q1 to Q3 and the whiskers indicate variability outside of Q1 and Q3 (*n* = 200). The statistical differences, according to the Mann–Whitney test, were as follows: * (*p* < 0.05), *** (*p* < 0.001). Scale bar (**A**) = 0.5 cm; (**B**,**D**) = 20 μm**,** (**C**) = 10 μm; (**E**) = 5 μm.

The thallus was soft, expanded, thick and flat, with long, 60 cm fluffy outgrowths, like a squirrel’s tail, or tuft-like (bushy) and up to 15 cm in length; purple to red-brown (Figure 2C; Figure S1). Fascicles contained a maximum of ten filaments, intertwined or joined together, parallel, by agglutinating sheaths, and often bifurcate (Figure 3A–C; Figure 4D,E). Sheaths were colourless, open at the ends, mostly thin and smooth, but sometimes thick, diffluent at the margin or lamellated, sporadically widened or had transverse folds (Figure 3D–H). Trichomes were long, straight, sometimes narrowed towards the end, purple or pink in colour, either unconstricted or slightly constricted at the cross-walls and enclosed in an individual sheath.

Cell width was 7–11.6 µm, and cell length was 4.1–11 µm (Figure 4F). The shape of the apical cell was polymorphic, being usually obtuse or rounded, sometimes elongated rounded–conical, without calyptra (Figure 3D–H and Figure 4E). The degree of granulation varied from being indistinct to very dense; in older trichomes, cell content kerithomised. Reproduction occurred through the disintegration of the trichome by the necridia into small fragments, known as the hormogonia (Figure 3B). The germination of the hormogonium next to the maternal trichome led to false branching of the coleodesmoid type (Figure 3B). Trichomes and hormogonia had gliding motility.

Strain BBK-W-15 was isolated from the fouling of a diseased sponge sampled near to the Bolshiye Koty settlement. In a liquid medium, the species grew as a thin biofilm and then formed spherical rhizoidal colonies up to 7 mm in diameter (Figure 4A), which were similar to the photogranules observed in the lake plankton (Figure 2E). Sheaths were thin and sometimes absent. Trichomes were straight, with slight constrictions, and sometimes narrowed towards the end; the shape of the apical cell was polymorphic, but most often, it was rounded or obtuse conical (Figure 4C). Cells were nearly isodiametric, 7–13.4 µm in diameter and 3–10.7 µm in length; the minimum and maximum lengths refer to cells that had recently divided or were about to divide. Overall, with the same mean trichome width of 9.4 µm (SD of strain = 1.2, SD of natural samples = 0.7), the strain was distinguished by trichomes that were wider than the natural samples and had a slightly lower mean cell length of 6.4 µm compared to 7 µm (SD of strain = 1.6, SD of natural samples = 1.5). Although cell size parameters differed significantly between the culture and natural samples, according to the Mann–Whitney test, these differences were small (Figure 4F).

**Figure 5:**
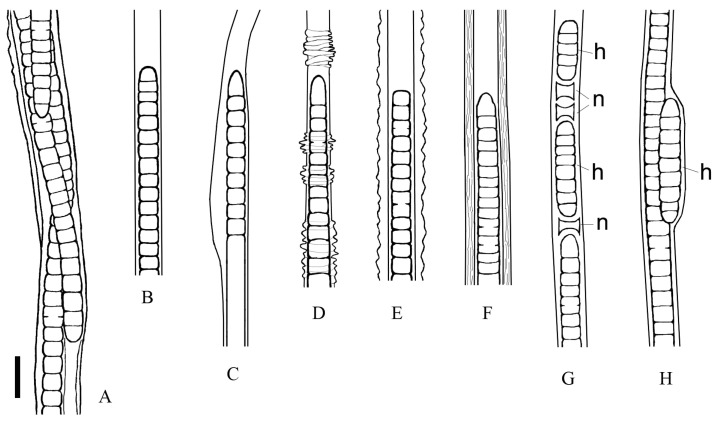
Morphology of fascicles and filaments of *Limnofasciculus baicalensis* gen. et sp. nov. according to microscopies of strain and natural samples. (**A**) A fascicle consisting of filaments agglutinated together by sheaths. (**B**–**F**) Different types of apical cell (a. c.) and sheath (s.) that occur randomly: rounded a. c., thin s. (**B**); acute conical a. c., thin widened s. (**C**); gradually attenuated trichome with rounded conical a. c., transversely folded s. (**D**); flat a. c., thick, diffluent at the margin s. (**E**); rounded conical stepped a. c., thick, lamellated s. (**F**). (**G**) Formation of hormogonia by means of necridic cells. (**H**) Germinating hormogonium within the mother sheath. Designations: h—hormogonium, n—necridic cell. Scale bar = 20 μm.

The morphological features of the species, which were based on microscopy of natural specimens and strain, are summarised and shown in Figure 5. The characteristics that distinguished this species from previously described morphologically similar forms (*Schizothrix* sp. Izhboldina and *Symplocastrum penicillatum* (Gom.) Anagn.) are listed in Table 1.

### 3.3. Cell Ultrastructure

**Figure 6:**
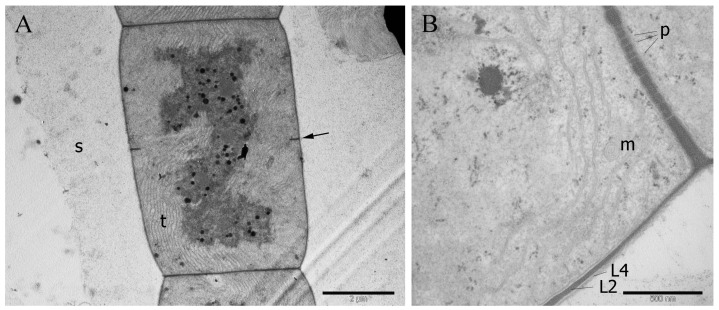
Transmission electron micrograph strain showing fascicular thylakoids and type C cell division (**A**). The structure of the cell wall with a thickened peptidoglycan layer and intercellular (junction) pores (**B**). Designations: arrow—dividing cell transverse septum; s—a layer of slime not framed in a sheath; t—thylakoids; m—mesosome; L2—peptidoglycan layer of the cell wall; L4—outer membrane, p—junction pores. Scale bar (**A**) = 2 μm; (**B**) = 0.5 μm.

Fascicular thylakoids in the longitudinal section were identical in appearance to the radial thylakoid pattern of cyanobacteria of the families Microcoleaceae (formerly Phormidiaceae) and Coleofasciculaceae (Figure 6A) [41,42]. The cell wall was four-layered, the peptidoglycan layer was two-thirds the thickness of the cell wall and the outer membrane was thin (Figure 6B). Cell division of the strain, in which daughter cells divided and reached maturity before the next division, was also a common feature of the above families, type C of Anagnostidis and Komárek [43]. Inclusions in the cytoplasm were represented by cyanophycin granules, polyphosphate granules, polyhedral bodies and lipid granules near the cell walls.

### 3.4. Molecular and Phylogenetic 16S rRNA Gene Analyses

A BLAST search of the 16S rRNA gene sequence of strain *Limnofasciculus baicalensis* gen. et sp. nov. using the NCBI GenBank database indicated that BBK-W-15 was closely related to three strains isolated from freshwater streams in Hawaii, USA, which were labelled as Oscillatoriales cyanobacterium HA4819-PD1 (GenBank accession KC525083, pairwise identity 99.7%), Oscillatoriales cyanobacterium HA4803-PD1 (KC525084, 99.6%) and *Phormidium* sp. 00767_00001 (KC854786, 99.5%). Other closest relatives had a low percentage of homology (94.5%) and were represented by *Coleofasciculus chthonoplastes* strains WW1 and WW2, which were isolated from a windy and flat area of the Baltic Sea coast, Germany [44]. The similarity between the BBK-W-15 16S rRNA gene sequence and the previously obtained sequences of uncultured clones from benthic fouling of Lake Baikal (GenBank accession KX348289 and KX348290 [9]) was 99.9%.

**Figure 7:**
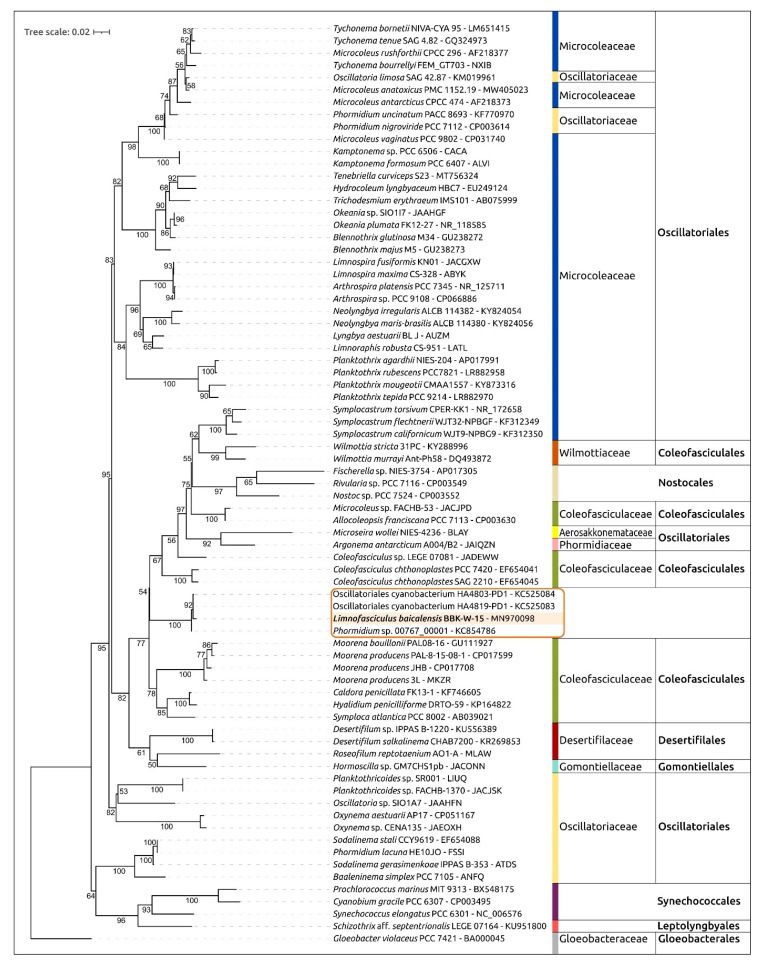
Best-scoring ML phylogenetic tree constructed using 75 16S rDNA nucleotide sequences, including the sequences most similar to 16S rDNA of *Limnofasciculus baicalensis* gen. et sp. nov. BBK-W-15 and representative sequences of different cyanobacterial taxa. *Gloeobacter violaceus* PCC 7421 was used as an outgroup. The GenBank accession is shown to the right of the organism’s name. The numbers near the tree branches indicate the TBE support. The total number of bootstrap trees was 1000. The scale bar shows 0.02 estimated substitutions per site. Complete and nearly complete rDNA sequences of rDNA BBK-W-15 (1486 bp) and the closest classified species were used to construct the tree, paying particular attention to this factor. The minimum rDNA length in the alignment was identified for *Trichodesmium erythraeum* IMS101 (1147 bp), and 64 out of 75 sequences were longer than 1300 bp.

The 16S rDNA phylogenetic tree constructed using BBK-W-15 and representative sequences, including the 16S rDNA sequences of Hawaiian cyanobacteria, placed the BBK-W-15, HA4803-PD1, HA4819-PD1 and 00767_00001 sequences in a monophyletic branch, which was distant from other genera and adjacent to representatives of the Coleofasciculaceae family (Figure 7). Cyanobacteria of the genus *Symplocastrum* isolated from the desert soils of North America also formed a distinct clade.

### 3.5. 16S-23S ITS Secondary Folding Structure Analysis

**Figure 8:**
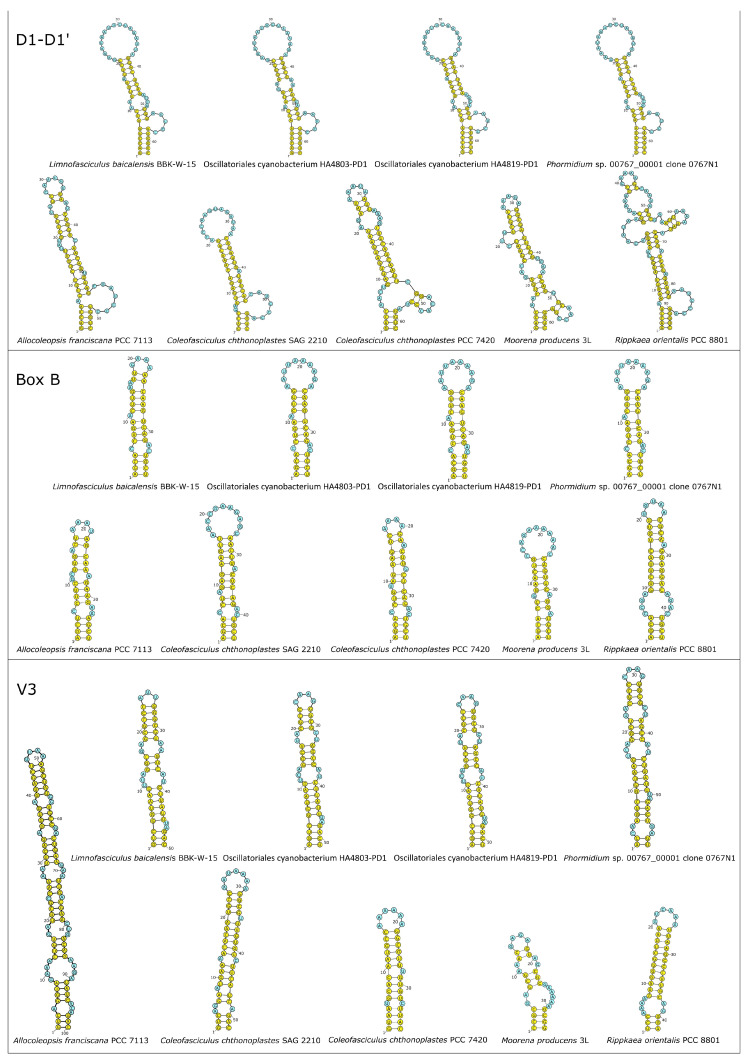
D1-D1′, Box B and V3 helices from *Limnofasciculus baicalensis* gen. et sp. nov. BBK-W-15 and other most similar representative 16S-23S ITS sequences. Yellow circles indicate paired nucleotide bases, and blue circles indicate unpaired bases. Numbers denote the nucleotide position, which starts from the 5′-end of the helix.

Available 16S-23S ITS regions of *Limnofasciculus baicalensis* gen. et sp. nov. BBK-W-15, the three above-mentioned Hawaiian freshwater cyanobacteria (Oscillatoriales cyanobacterium HA4803-PD1, Oscillatoriales cyanobacterium HA4819-PD1 and *Phormidium* sp. 00767_00001) and the most similar sequences, according to the results of sequence comparisons and phylogenetic analysis (*Allocoleopsis franciscana* PCC 7113, *Coleofasciculus chthonoplastes* SAG 2210, *C*. *chthonoplastes* PCC 7420, *Moorena producens* 3L and *Rippkaea orientalis* PCC 8801), were used for the secondary folding structure analysis (Figure 8). The ITS D1-D1′ helices of BBK-W-15 and Hawaiian strains HA4803-PD1, HA4819-PD1 and 00767_00001 had very similar sizes and overall secondary structures, though they showed some differences in terms of bilateral bulges. The *Phormidium* sp. 00767_00001 D1-D1′ helix folding structure was the closest to the corresponding BBK-W-15 structure. The folding structure of D1-D1′ helices of BBK-W-15 and Hawaiian strains showed distinct differences to other cyanobacterial strains, both in terms of bilateral bulges and terminal loops.

The Box B helices of *Limnofasciculus baicalensis* gen. et sp. nov. BBK-W-15 and the three Hawaiian cyanobacteria had nearly identical sequences and similar basal regions, though they differed in terms of terminal loops because of single nucleotide G → A substitution in the BBK-W-15 terminal loop (compared to the terminal Box B loops in Hawaiian cyanobacteria), resulting in a reduction in the size of the loop from 10 to 4 nucleotides. The Box B helix of BBK-W-15 and the Hawaiian strains differed from other strains in terms of bilateral bulges and the sequence of the terminal loop, though they were similar in terms of the basal clamp part of the helix. The V3 helix was characterised by greater variability. The closest V3 helices to BBK-W-15 were in Oscillatoriales cyanobacteria HA4803-PD1 and HA4819-PD1. The V3 helix of *Phormidium* sp. 00767_00001 differed from BBK-W-15 V3 helix in the basal part, though it was similar in the remaining part. Four V3 helices of BBK-W-15 and Hawaiian cyanobacteria showed distinct differences in terms of secondary folding structure compared to V3 helices in other strains.

### 3.6. Average Nucleotide Identity Calculations of Concatenated Conserved Protein Sequences’ Phylogeny, Genome Statistics and Proteome Analysis

The heterogeneity score of the resulting genomic assembly, which was assessed via CheckM, was 0.00, while the completeness score was 97.15, and the contamination score was 1.41. Calculations of average nucleotide identity (ANI) were conducted using the genomic sequences of *Limnofasciculus baicalensis* gen. et sp. nov. BBK-W-15 and a total of 1614 genomes deposited in GenBank as of April 2023. The highest ANI value of 73.19% was found for the genomic sequence of *Coleofasciculus* sp. LEGE07092. The clustered heat map was calculated using the genomes of 150 strains, including strains closest to BBK-W-15 and representative genomic sequences of cyanobacteria belonging to different taxonomic groups, showed significant differences between BBK-W-15 and other cyanobacteria genomes (Table S2). Unfortunately, the genomes of Hawaiian freshwater cyanobacteria Oscillatoriales cyanobacterium HA4803-PD1, Oscillatoriales cyanobacterium HA4819-PD1 and *Phormidium* sp. 00767_00001 were not sequenced; thus, it is impossible to estimate their ANI values.

**Figure 9:**
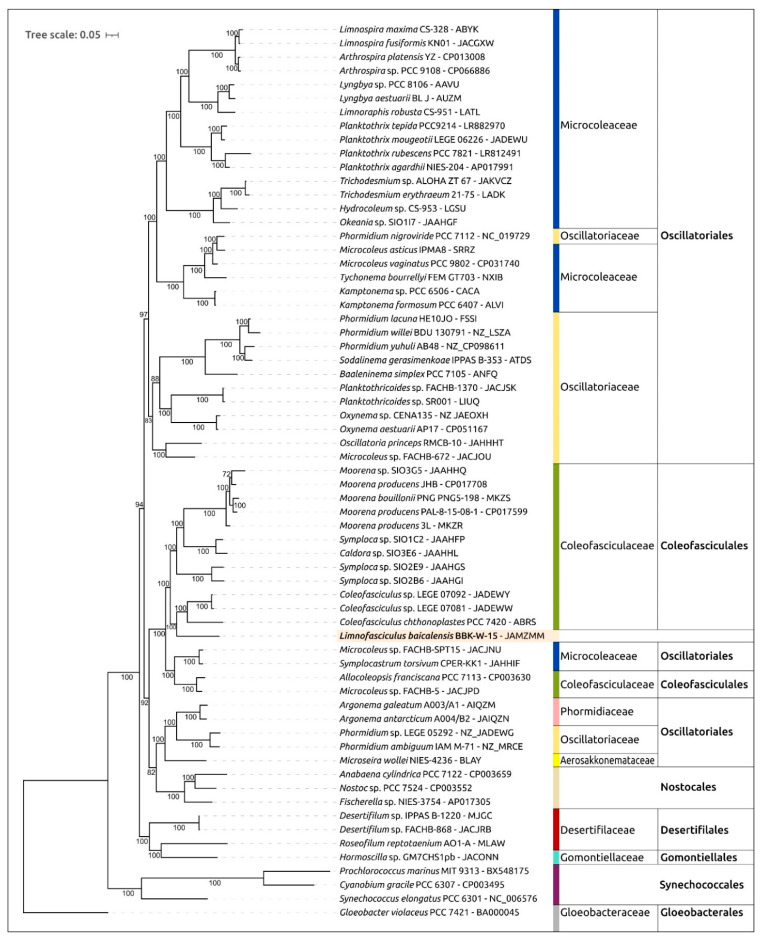
Best-scoring ML phylogenetic tree constructed using 65 concatenated amino acid sequences of 400 conserved proteins of *Limnofasciculus baicalensis* gen. et sp. nov. BBK-W-15 and representatives of different cyanobacterial taxa. *Gloeobacter violaceus* PCC 7421 was used as an outgroup. The GenBank accession is shown to the right of the organism’s name. The numbers near the tree branches indicate the TBE values. The total number of bootstrap trees was 100. The scale bar shows 0.1 estimated substitutions per site.

Phylogenetic analysis using a concatenated alignment of amino acid sequences was conducted using the PhyloPhlAn pipeline, which employs the 400 most conserved proteins to obtain an alignment. A total of 65 strains, including *Limnofasciculus baicalensis* gen. et sp. nov. BBK-W-15, as well as the closest genomes found using ANI calculations and genomic sequences belonging to different cyanobacterial groups, were used for the analysis (Figure 9). This tree showed greater resolution and bootstrap support than the 16S rDNA phylogenetic tree. The tree placed BBK-W-15 distantly from other groups, in a singleton branch belonging to a large clade that includes representatives of Coleofasciculaceae. The genetic distance between BBK-W-15 and the closest cyanobacteria was of the same order as that between different cyanobacterial genera of the same family. Based on the phylogenetic analysis, it can be concluded that *Limnofasciculus baicalensis* gen. et sp. nov. is a new genus of the family Coleofasciculaceae. Interestingly, in both the concatenated conserved protein sequences’ phylogenetic tree and the 16S rDNA tree, some families, including Coleofasciculaceae, were not monophyletic.

**Figure 10:**
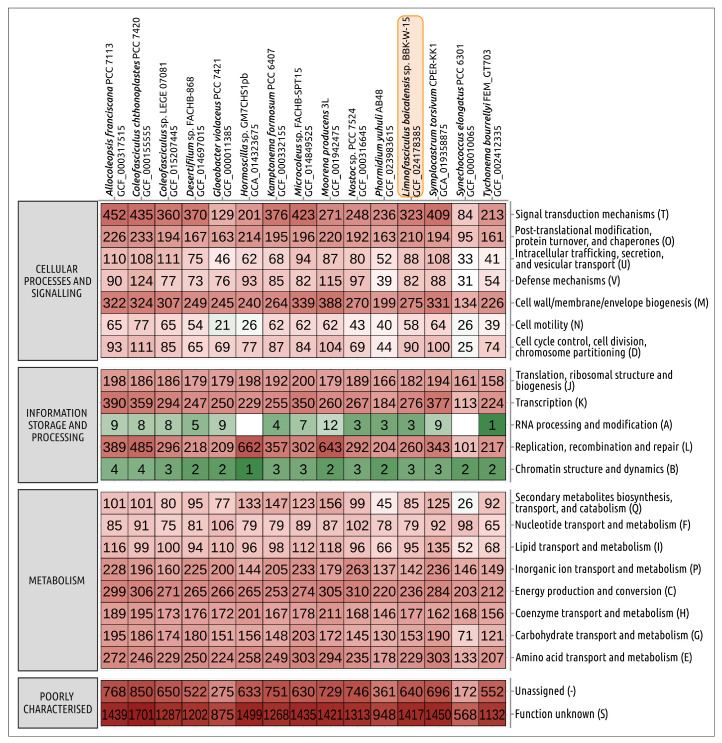
Heatmap showing the distribution of clusters of orthologous groups of proteins belonging to *Limnofasciculus baicalensis* gen. et sp. nov. BBK-W-15 and other cyanobacteria obtained using eggNOG-mapper. The numbers displayed in cells indicate the number of proteins belonging to the orthologous groups.

The size of the draft genomic assembly of *Limnofasciculus baicalensis* gen. et sp. nov. BBK-W-15, calculated as the size of genomic contigs, was 6,604,967 bp, which was close to the size of the genomic assembly of *Coleofasciculus* sp. LEGE07092 (6,712,583 bp). The G + C content in the genomic DNA of BBK-W-15 was 42.0%, which was slightly lower than that of *Coleofasciculus* sp. LEGE07092 (46.1%), but within the range of normal values of cyanobacteria. The number of predicted protein coding sequences was 5710, which was close to that of *Coleofasciculus* sp. LEGE07092. A comparison of the distribution of clusters of orthologous groups of proteins (COGs) indicated similarities in distribution between BBK-W-15 and phylogenetically related strains (Figure 10). No uniqueness in the distribution of clusters of orthologous groups of proteins was revealed via the analysis.

### 3.7. Biosynthetic Potential of Limnofasciculus Baicalensis

**Figure 11:**
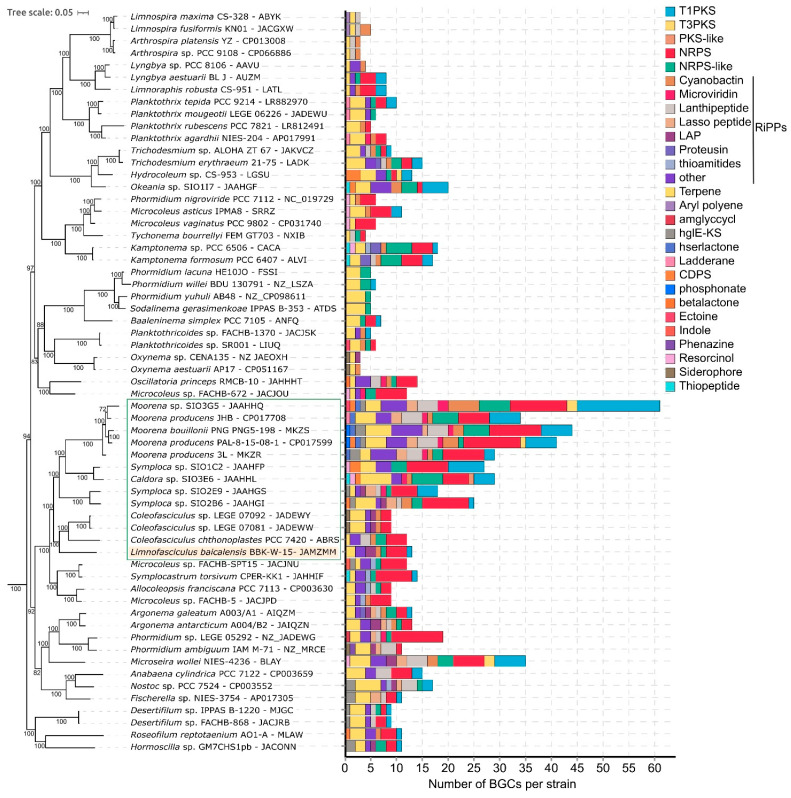
Composition of BGCs identified in the genomes of *Limnofasciculus baicalensis* gen. et sp. nov. BBK-W-15 and other filamentous cyanobacteria. The absolute number of BGCs per strain assigned to each BGC class, according to the antiSMASH classification, is shown. The Coleofasciculaceae cluster is delineated on the phylogenetic tree, with tree parameters defined as shown in Figure 9.

Benthic cyanobacterial strains, especially those associated with sponges and corals, possess a wide variety of biosynthetic gene clusters (BGCs) that encode biologically active substances. The genomic repertoire of the secondary metabolism of the *Limnofasciculus baicalensis* gen. et sp. nov. genome was predicted using antiSMASH (Figure 11). The biosynthetic capabilities of BBK-W-15 were compared to the chemically rich marine cyanobacteria of the family Coleofasciculaceae and the closest related strain *C. chthonoplastes* PCC 7420 (Table S3). Most of the BGCs in the BBK-W-15 genome were predicted to encode ribosomally synthesised and post-translationally modified peptides (RiPPs) (5 BGC), as well as non-ribosomal peptide synthetases (NRPS) (4 BGC), followed by polyketide synthases type I (T1PKS) and terpenes (2 BGC each). In the genome of *Limnofasciculus*, there were BGCs encoding substances such as geosmin, which is a strong odorant that deteriorates drinking water quality; enediyene, which is an anticancer antibiotic; microcyclamide (cyanobactin), which is a cytotoxic cyclic hexapeptide; varlaxins, which are new aeruginosin-type inhibitors of human trypsins; 1-heptadecene, which is an unsaturated aliphatic hydrocarbon considered as a potential biofuel; and rhizomides, which form a group of cyclic xenopeptides, and a linear azol(in)e-containing peptides with antibacterial activities.

Overall, benthic cyanobacteria had the broadest spectrum of BGCs on the tree, with the marine tropical *Moorena* spp. having the highest number of BGCs, which ranged from 26 to 61 (Figure 11). Planktic cyanobacteria generally had the lowest number of BGCs per strain (3-5 BGCs). In the cluster of cyanobacteria of the family Coleofasciculaceae outlined in the tree, chemically rich strains of the tropical marine cyanobacteria *Caldora*, *Moorena* and *Symploca* contained two to five times more BGCs in their genomes than *Limnofasciculus baicalensis* and related *Coleofasciculus* sp. strains.

### 3.8. Taxonomic Treatment

***Limnofasciculus*** Sorokovikova and Tikhonova, **gen. nov.**—TYPE: *Limnofasciculus baicalensis*.

*Etymology: Limnofasciculus* is derived from *“*Lim.no.fas.’ci.cu.lus” in which Gr. fem. n. *limnis* means lake; L. masc. n. *fasciculus* is fascicle, bunch; and in general N.L. masc. n. *Limnofasciculus* is a freshwater cyanobacterium with trichomes arranged in fascicles.

*Description: Limnofasciculus* is related to Microcoleaceae (Oscillatoriales) and Coleofasciculaceae (Coleofasciculales) with trichomes in sheaths (*Symplocastrum* and *Coleofasciculus*), but it differs in its lifestyle, as it is benthic in freshwater lake and genome, as determined via comprehensive analyses, including phylogenetic studies. 

Thallus tuft-like, ascending, pseudobranched or thick and expanded. Filaments with one trichome in open sheath agglutinated into fascicles. Sheaths colourless, firm, sporadically with transverse striations or lamellated. Trichomes straight, slightly constricted at the cross walls, with type C cell division and gliding motility. Cells near to isodiametric, with radial or fasciculated thylakoid arrangement. Apical cells rounded to elongated, conically rounded, noncalyptrate. Reproduction by fragmentation of trichomes into motile hormogonia through the help of necridic cells. The main reason for its isolation is its unique and distinct position, as determined via a comprehensive analysis, including 16S rRNA gene phylogeny, conserved protein phylogeny and whole-genome comparisons. Its habitat is freshwater, nonterrestrial.

***Limnofasciculus baicalensis*** Sorokovikova and Tikhonova, **sp. nov.**—HOLOTYPE: Lake Baikal, Bolshiye Koty settlement, Russia (February 2015; 51.90203° N 105.10561° E). A portion of a culture of *Limnofasciculus baicalensis* BBK-W-15 was preserved in metabolically inactive form in the Herbarium of Irkutsk State University (IRKU), the Department of Botany and Genetics, Irkutsk State University, Irkutsk, Russia, and is available under the accession number IRKU092121. The reference strain was deposited in the collection of microalgae and cyanobacteria IPPAS, the Timiryazev Institute of Plant Physiology, the Russian Academy of Sciences, Moscow, Russia, under the accession number IPPAS B-2062. The genome data are available at DDBJ/EMBL/GenBank under the accession number JAMZMM000000000.

*Etymology*: The species name is pronounced “bai.ca.len.’sis”. N.L. masc. adj. *baicalensis* is named in honour of Lake Baikal, which is the location where the type strain was isolated.

*Description:* Thallus formes purple to red-brown mat on agar or flask wall, in a liquid medium aggregates into spherical rhizoid colonies. In nature, the thallus morphology ranges from tuft (1–15 cm long) to extensive mats with long outgrowths (up to 60 cm); attached to stones, sand, sponges, submersed wood and other objects, rarely found in photogranules in plankton. Fascicles contain one to 10 filaments intertwined or joined in parallel by agglutinating sheaths, often bifurcate. Filaments with one trichome. Sheaths open, colourless, thin, firm (sporadically widened, with transverse striations, sometimes thick, lamellated or diffluent at the margin) (Figure 5). Trichomes straight, not (or slightly) constricted at the cross walls, with gliding motility, 7–13.4 μm wide. Cells isodiametric or shorter than wide, 3–11 μm long, sometimes with fine granulation in the centroplasm, with type C cell division, thylakoids fasciculated. Apical cell rounded to elongated rounded-conical, sometimes flat or stepped with nipple. Reproduction by motile hormogonia formed by necridia. Germination of hormogonium beside the maternal trichome leads to false branching of coleodesmoid type (Figure 5).

*Diagnosis:* This species is morphologically similar to the cyanobacteria of the genus *Symplocastrum* and *Coleofasciculus*. The main distinguishing morphological features are the red-brown colour and large size (up to 60 cm) of the tuft thallus, one trichome in filament, colourless sheaths with transverse striations, isodiametric or short cells and false branching of coleodesmoid type. Ecological differences consist of a benthic freshwater lifestyle, a variety of substrates for attachment (from inanimate objects to branched and encrusting sponges) and living in plankton in the form of photogranules. The 16S rRNA gene phylogeny and conserved protein phylogeny demonstrate that the strain BBK-W-15 shares a monophyletic branch with representatives of the Coleofasciculaceae family, but its location is distant from representatives of other known genera of this family (Figure 9). Analysis of the secondary structures of the 16S-23S ITS region of strain BBK-W-15 and related cyanobacterial strains shows the characteristic features of this region and indicates notable differences from other phylogenetically related taxa (Figure 8).

*Habitat:* freshwater.

*Distribution area:* Lake Baikal, Russia.

## 4. Discussion

Global climate warming is thought to be a major cause of harmful cyanobacterial blooms in lakes [45,46]. Benthic cyanobacteria also respond to changing environmental conditions, and their proliferation in aquatic ecosystems is often associated with the deterioration in water quality, the death of sponges and corals and the poisoning of domestic animals with cyanotoxins [47,48,49].

In Lake Baikal, the annual temperature has risen at an average rate of 1.2 °C per 100 years since 1896. Over the past 60 years, the temperature of the upper layers of the water has gradually increased during the warm season [50]. There has also been an increase in nutrient concentrations in the lakeshore area and in the groundwater of beaches near settlements due to inadequate wastewater treatment [51,52,53]. The growth of tourism has also led to increased concentrations of nutrients in the lake water area due to anthropogenic pressure [54,55].

Changing ecological conditions have probably induced the proliferation of the species, the occupation of new ecological niches (sponges, sand, submerged objects) and a change in its morphology with an increasing trichome size (1.4-fold) and thallus size (10- to 60-fold) (Table 1). According to surveys of phytobenthos conducted throughout the Baikal area in the period 1961–1985, *Schizothrix* sp. (morphologically similar to the *Limnofasciculus baicalensis* gen. et sp. nov.) was found in vegetation zones 1–3 at depths of 1.5–15 m, and it was present all year round on rocks and rarely on sand or among thalli of the green algae *Cladophora* spp. The species has never been recorded on Baikal sponges [3]. The use of the sponge body as a substrate for the growth of filamentous cyanobacteria was previously described in some diseased marine sponges [56,57], with this phenomenon not being common in freshwater ecosystems, except for Lake Baikal. The growth of filamentous cyanobacteria on coral reefs, causing disease and degradation, has been more frequently reported in seas around the world [48,58,59]. This finding means that, in marine ecosystems, the formation of visible cyanobacterial mats and fouling on animals (sponges and coral polyp colonies) is always associated with disease and destruction of the latter species, as occurred in Lake Baikal [8,9,11].

The ability of *Limnofasciculus baicalensis* gen. et sp. nov. to form photogranules is not unique, though it has only been described for only a small number of species and is unusual for lacustrine cyanobacteria. In nature, cyanobacterial photogranules have only been described in cryoconites from glaciers located in Greenland, the Arctic and the Tien Shan Mountains, China. The cryoconite photogranules were formed by the thin filamentous cyanobacteria *Phormidesmis priestleyi*, *Tychonema* sp. and *Leptolyngbya* sp. around a core of mineral particles, and they were no more than 1.4 mm in diameter [60,61,62]. Larger photogranules, which measured up to 2 cm in diameter, were formed by *Microcoleus* sp. and *Tychonema* sp. in wastewater treatment systems [63,64]. The reasons for the formation of photogranules in such diverse and specific ecosystems are not yet fully understood, though it is known that mobile filamentous cyanobacteria play a leading role in this process. It is expected that the use of oxygenic photogranules will reduce sludge aeration and, by closing the CO_2_ and O_2_ cycles, help to create a renewable biological raw material from waste water [65]. In Lake Baikal, the occurrence of cyanobacterial photogranules, as well as algal balls, is probably related to the hyperproduction of benthic cyanobacteria and algae, and serves as a way of self-purification of the ecosystem [14,66,67]. The possible biotechnological potential of the strain requires further investigation.

Morphologically, the new species combines features of the representatives of several genera in the orders Oscillatoriales and Coleofasciculales. Thick, lamellated sheaths and those with occasional transverse folding are found in the genera *Symplocastrum*, *Lyngbya* and *Blennothrix* (Microcoleaceae family) [19,68]. The coleodesmoid type of false branching relates to the species of the genus *Blennothrix* [69]. Bushy colonies, sheath polymorphism and isodiametric cells are also common in *Symplocastrum californicum* and *S*. *flechtnerae* [21]. Trichome morphology is also similar to *Coleofasciculus*; the colour and habitus of the thallus; fascicles with parallel trichomes, with one trichome in the sheath; and the fouling of benthic attached animals are characteristic of the species *Symploca*, *Caldora* and *Moorena* (Coleofasciculaceae family) [70,71,72]. The ultrastructural features of the strain are also common in both cyanobacterial orders and do not clarify its taxonomic position. Identification of cryptic genera with such unclear morphology is only possible when using molecular methods [16].

Previous meticulous work carried out on 3500 genomes that represented type strains of species from more than 850 bacterial or archaeal genera revealed that the ANI values of the prokaryotic genus demarcation threshold have a mean of 73.98% (25% quartile, 70.85%; 75% quartile, 76.56%) [73]. By applying the recommended threshold for bacterial genera demarcation (83%) [74], as well as conducting regular practice of new genera delineation, the isolation of the new genus *Limnofasciculus* can be confidently proposed based on the determined ANI values. Based on phylogenetic analysis and other molecular data, this novel genus can be assigned to the family Coleofasciculaceae of the order Coleofasciculales. Originally, the family contained desiccation-resistant filamentous cyanobacteria, which were mainly terrestrial and occured from tidal zones [75]. The marine benthic cyanobacteria of the genera *Symploca*, *Caldora* and *Moorena*, which were mostly associated with corals, were also included in the new extended family description [68]. The appearance of a new genus of freshwater lacustrine benthic cyanobacteria within Coleofasciculaceae broadens the ecological characterisation of the family.

Cyanobacteria are known producers of a wide range of bioactive metabolites [76]. A search for biosynthetic gene clusters in the *Limnofasciculus* genome showed that it is inferior to marine tropical cyanobacteria in terms of BGCs number, though it can be a producer of a range of interesting substances with anticancer (enediyene and microcyclamide) and antibacterial (rhizomides and linear azol(in)e-containing peptides) activities. The production and release of antibiotics into the environment gives the species a competitive advantage within the benthic community. Mass development of the species can degrade drinking water quality due to its excretion of the odorant geosmin. Plasmid studies can extend these findings, as only 95% of natural product BGCs are based in the genome of cyanobacteria, as described in [77].

Owing to the widespread availability of metagenomic studies, cyanobacteria have been shown to have a more cosmopolitan distribution, and endemic taxa are less common than previously suggested [78]. The “everything is everywhere” hypothesis has been confirmed for microbial biogeography [79]. Research into algal and cyanobacterial biodiversity in Hawaii revealed a great diversity of freshwater and terrestrial cyanobacteria, many of which have been characterised as endemic [80,81]. However, the phylogenetic analysis of 16S rDNA closely related to the Baikalian strain revealed that the newly established genus *Limnofasciculus* may include Oscillatoriales cyanobacterium HA4803-PD1, Oscillatoriales cyanobacterium HA4819-PD1 and *Phormidium* sp. 00767_00001 that originates from Hawaiian freshwater streams. By applying the threshold for species demarcation using the nucleotide identity of 16S rRNA sequences of 98.5% [73], as well as conclusions from the analysis of the secondary structures of the 16S-23S ITS region, the latter strain could probably also be classified as *Limnofasciculus baicalensis* gen. and sp. nov., though more detailed analysis of its genome is required. Thus, the new genus of benthic cyanobacterium revealed in Lake Baikal is not endemic.

## Figures and Tables

**Table 1:** Morphological comparison of *Limnofasciculus baicalensis* gen. et sp. nov. with *Schizothrix* sp. Izhboldina and *Symplocastrum penicillatum* (Gom.) Anagn.

	*Limnofasciculus baicalensis* gen. et sp. nov. (*Symplocastrum* sp.)	*Schizothrix* sp. [3]	*Symplocastrum penicillatum* [19]
Thallus	1–15 (60) cm, purple to red-brown, without incrustation. Fascicles with 1–10 agglutinated filaments enclosed in individual sheaths, ascending, often pseudobranched	0.2–1 cm, blue-green, without incrustation. Filaments with 1–10 trichomes in a common sheath, aggregated to ascending fascicles, often pseudobranched	Dark green, dull blue-green, later incrusted, yellowish. Filaments with 1–5 trichomes, at the base entangled, at the ends parallel aggregated to ascending, penicillate fascicles, not (or sparsely) pseudobranched
Trichome width	7–11.6 (13.4) µm	6.6–8.3 μm	2.4–5 (6) μm
Cell length	(3) 4.1–11 µm	6–8.3 (13.5) μm	2–9 μm
Filament width with one trichome	12–15 (25) μm	11–13 μm	No data
Sheath	1–7.5 μm width. Sheaths open, colourless, firm, thin, sporadically widened or transversely folded, sometimes thick, lamellated or diffluent at the margin	2.7–5 μm width. Firm, colourless or yellowish, not lamellated, thin	Firm, colourless, not (or sporadically) lamellated, thick in the basal part, thin in the upper part
Apical cell	Rounded, elongated rounded–conical, sometimes flat or stepped with nipple	Rounded, rounded–conical	Obtuse–conical, rounded–conical
Occurrence	Along most of the Baikal coast at depths of 3–10 m, rarely up to 20 m, on rocks, sand, encrusting and branched sponges, fishing nets, wood, steel. In autumn, after storms, in plankton, aggregated into 0.5–1.5 cm photogranules	Along most of the Baikal coast at depths of 1.5–15 m, rarely up to 40 m, on rocks, less often on sand. Often grows among *Tolypothrix distorta* and *Cladophora* spp. thalli	Freshwater, in flowing waters, waterfalls and in stony littoral of clear mountain lakes, among mosses

## Supplementary Materials

The following supporting information can be downloaded via the following webpage: https://www.mdpi.com/article/10.3390/microorganisms11071779/s1. Figure S1: Morphology of thalli of the cyanobacterium *Limnofasciculus baicalensis* gen. et sp. nov. in Lake Baikal, Russia, near the Listvyanka settlement. Mats with squirrel-tail outgrowths on rocks and encrusting sponges; Table S1: NCBI accessions shown in Figure 7; Table S2: Clustered heatmap based on the calculations of ANI of the cyanobacterium *Limnofasciculus baicalensis* gen. et sp. nov. BBK-W-15 (under number 45) and representatives of other cyanobacterial taxa; Table S3: Biosynthetic gene clusters in *Limnofasciculus baicalensis* genome and the related cyanobacteria of the family Coleofasciculaceae (antiSMASH results).
