# Supplementary material for: Limnofasciculus baicalensis gen. et sp. nov. (Coleofasciculaceae, Coleofasciculales): A New Genus of Cyanobacteria Isolated from Sponge Fouling in Lake Baikal, Russia
Source: Microorganisms. 2023 Jul 9;11(7):1779. doi: 10.3390/microorganisms11071779 (PMC10385159; doi:10.3390/microorganisms11071779)
Supplement: Supplementary file 1 [file microorganisms-11-01779-s001.zip › Supplementary.pdf]

Supplementary Materials for Article

Limnofasciculus baicalensis gen. et sp. nov. (Coleofasciculaceae, Coleofasciculales): A new genus of cyanobacteria isolated from sponge fouling in Lake Baikal, Russia

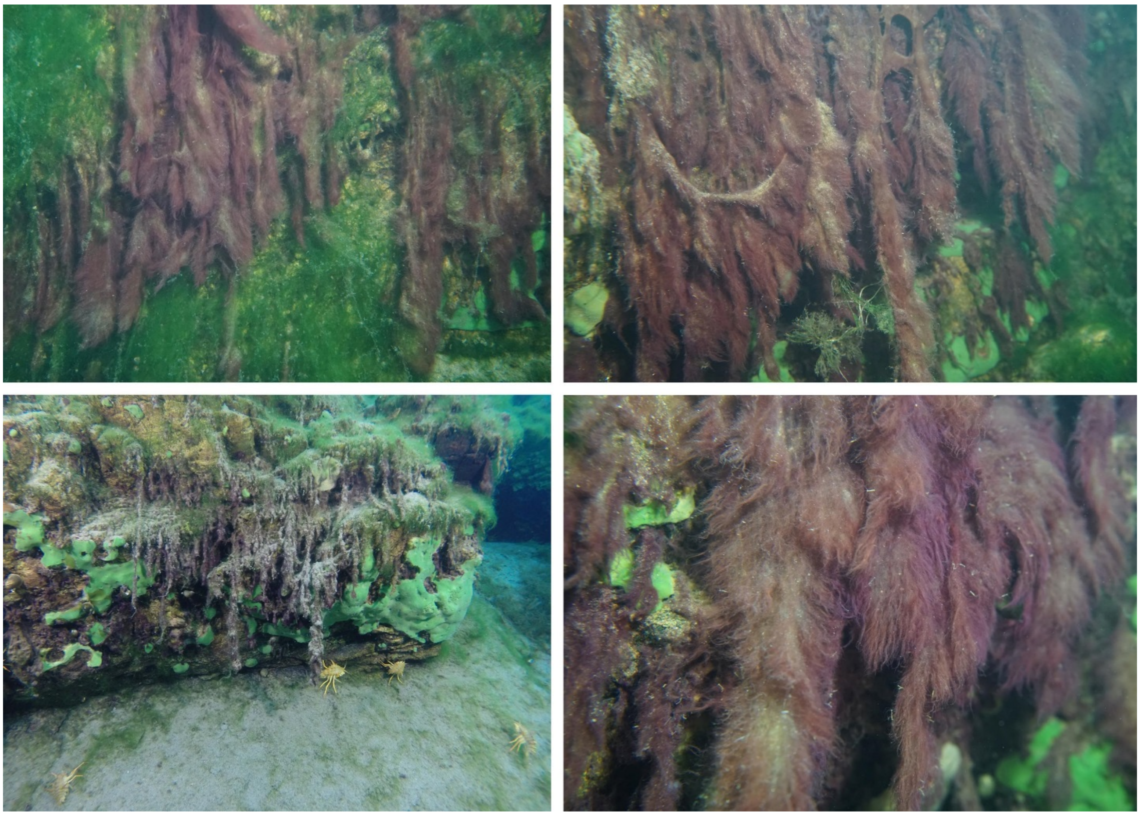

Figure S1. Morphology of thalli of the cyanobacterium *Limnofasciculus baicalensis* gen. et sp. nov. in Lake Baikal, near the Listvyanka settlement. Mats with squirrel-tail outgrowths on rocks and encrusting sponges.

Table S3. Biosynthetic gene clusters in *Limnofasciculus baicalensis* genome and the related cyanobacteria of the family Coleofasciculaceae (antiSMASH results).

Biosyn-thetic class	Compound	Number of gene clusters					
		L*	Cc	S	Mp	Mb	C
NRPS	anabaenopeptin 908, 915	-	-	1	-	1	-
	cyanochelin A	-	-	-	1	-	-
	desmamide A-C	-	-	-	1	-	-
	enediyene	1	-	-	-	-	-
	hexose-palythine-serine/hexose-shinorine	-	1	1	-	-	-
	kolossin	-	1	-	-	-	-
	microcystin	-	-	-	1	-	-
	micropeptin K139	-	-	1	-	-	1
	nostophycin	-	-	-	1	-	-
	octapeptin C4	-	-	-	-	-	1
	rhizomide A, B, C	1	-	-	1	-	-
	scytocyclamide A, B, C, B3, A2, B2	-	-	-	-	1	-
	vatiamide A-F	-	1	-	2	2	1

	varlaxin 1046A, 1022A	1	-	-	-	-	-
	vioprolide A-D	-	1	-	-	-	-
	unknown	1	2	1	3	4	6
Type I PKS	Curacin A	-	-	-	-	-	1
	1-heptadecene	1	-	-	1	1	1
	heterocyst glycolipids	-	-	1	-	-	-
	nostoclide N1, N2	-	-	1	-	-	-
	puwainaphycin A, B, C, D	-	-	-	1	1	-
	unknown	1	-	-	-	-	-
RiPPs	apratoxin A	-	-	-	-	2	-
	avilamycin A, C	-	-	-	-	-	1
	cyanobactin (microcyclamide/piricyclamide)	1	1	-	1	-	1
	Class II lanthipeptides like mutacin II (U40620)	-	1	1	1	-	-
	Glycosylated lanthipeptide/linaridin hybrids like MT210103	-	1	-	1	-	-
	Linear azol(in)e-containing peptide	2	1	2	-	1	1
	microviridin	-	-	1	1	1	1
	muscoride A, B	-	-	1	-	1	-
	mycosporine-like amino acids (MAAs)	-	-	-	2	-	-
	PcpA	-	-	-	1	-	-
	saxitoxin/neosaxitoxin/decarbamoylsaxitoxin/gonyautoxin3/ gonyautoxin 2	-	-	-	1	-	-
	unknown	2	-	4	3	5	-
Terpene	geosmin	1	-	-	-	-	-
	resorcinol	-	-	-	-	-	1
	unknown	1	1	1	4	2	3
Hybrid	anachelin	-	-	1	1	-	1
	anatoxin-a/homoanatoxin-a	-	-	1	-	-	1
	barbamide	-	-	-	1	-	-
	fischerazole A, B, C	-	-	-	-	1	-
	hectochlorin	-	-	-	-	1	-
	hapalosin	-	-	-	1	-	1
	jamaicamide A-C	-	-	-	1	-	-
	luminaolide	-	-	1	-	-	-
	minutissamide A, C, D	-	1	-	-	-	-
	nostopeptolide A2	-	-	1	-	-	1
	quartromicin A1	-	-	-	-	-	1
	tiacumicin B	-	-	-	-	1	-
	unknown	-	-	1	1	3	-
Other	ladderane	-	-	-	1	-	-
	homoserine lactone	-	-	-	1	1	-
**Total**		**13**	**12**	**21**	**34**	**29**	**24**

* L – *Limnofasciculus baicalensis* gen. et sp. nov. BBK-W-15, Cc – *Coleofasciculus chthonoplastes* PCC 7420, S – *Symploca* sp. SIO3G5, Mp – *Moorena producens* 3L, Mb – *Moorena bouillonii* PNG5-198, C – *Caldora* sp. SIO3E6.
